# The dual role of the ubiquitin–proteasome system in photomorphogenesis

**DOI:** 10.1042/EBC20253023

**Published:** 2025-10-28

**Authors:** Hua Zhou, Xing Wang Deng

**Affiliations:** 1 Laboratory of Plant and Environment Interaction Study, Tsientang Institute for Advanced Study, Hangzhou, China; 2 Peking-Tsinghua Center for Life Sciences, School of Advanced Agriculture Sciences and School of Life Sciences, Peking University, Beijing, China

**Keywords:** E3 ligase, photomorphogenesis, protein ubiquitination and degradation, the ubiquitin–proteasome system

## Abstract

The ubiquitin–proteasome system (UPS) represents a highly conserved protein degradation pathway that plays an essential role in maintaining the homeostasis of cellular proteins. This system ensures precise regulation of key regulators within the light signaling pathway, thereby enabling plants to dynamically switch between skotomorphogenesis (growth in the dark) and photomorphogenesis (growth in the light). In darkness, the negative E3 ligases (e.g. CRL4^COP1–SPA^) target photomorphogenesis-promoting regulators (e.g. ELONGATED HYPOCOTYL5) for ubiquitination and degradation, consequently repressing photomorphogenesis. Conversely, under light conditions, the positive E3 ligases (e.g. CRL1^EBF1/2^) promote the ubiquitination and degradation of photomorphogenesis-inhibitory regulators (e.g. phytochrome-interacting factors), ensuring proper seedling photomorphogenic development. This mini-review provides a concise overview of the ubiquitin-proteasome system in plants, focusing on recent advances in understanding the role of the UPS in regulating photomorphogenesis. Additionally, we highlight current challenges in further exploring the role of the UPS in photomorphogenesis.

## Introduction

Besides serving as a source of energy, light also functions as an essential signaling molecule that profoundly influences plant growth and development. After germination in the soil in darkness, plants employ the developmental program called skotomorphogenesis, forming an apical hook and accelerating their hypocotyl growth to successfully reach the soil surface before running out of the stored energy reserves [1]. Upon perceiving light, plants rapidly switch developmental programs from skotomorphogenesis to photomorphogenesis, resulting in dramatic physiological changes in the seedlings. The hypocotyl elongation of *Arabidopsis* seedlings is suppressed, while the cotyledons expand and green with chloroplasts, preparing for photosynthesis [1].

Light, one of the most critical environmental cues, induces broad transcriptional and translational reprogramming during photomorphogenesis [1–6]. At the transcriptional level, it is estimated that the expression of one-third of the *Arabidopsis* genome is under the control of light [1,6–8]. At the protein level, light enhances global translational efficiency in *Arabidopsis* by increasing ribosome occupancy and density on mRNAs [1–4]. In addition, light can reshape the translatome through inducing various post-translational modifications (PTMs) of proteins, such as ubiquitination and phosphorylation [2,9–11]. Among these PTMs, ubiquitination has been extensively studied and is considered essential for plant adaptation to changing environmental cues, including the dynamic fluctuations in light conditions [12].

The ubiquitin–proteasome system (UPS), which is a regulatory mechanism for selective protein degradation, plays a pivotal role in seedling photomorphogenic development [9,11,13]. In this review, we first give a brief introduction to the UPS in plants. Subsequently, we focus on the recent advances in understanding the role of the UPS in regulating photomorphogenesis. Additionally, we discuss the current challenges in further investigating the role of the UPS in photomorphogenesis.

### Overview of the ubiquitin–proteasome system in plants

The UPS, an essential protein degradation pathway consisting of five essential components which are ubiquitin, E1 (ubiquitin-activating enzyme), E2 (ubiquitin-conjugating enzyme), E3 (ubiquitin ligase), and the intact 26S proteasome, plays a vital role in maintaining cellular protein homeostasis [12,14]. The UPS is a two-step protein degradation pathway: first, ubiquitin molecules are covalently attached, typically to lysine residues of substrate proteins, and subsequently, poly-ubiquitin chain-attached proteins are degraded by the 26S proteasome [12,14]. In the initial step, E1 catalyzes the activation of ubiquitin in an ATP-dependent manner, and then the activated ubiquitin is transferred to a cysteine residue of E2, and eventually, E3 recognizes specific substrate proteins and mediates the transfer of the activated ubiquitin either from E2 or directly from itself to the substrate proteins [12,14]. In the second step, the 26S proteasome, a multi-catalytic protease complex, recognizes and degrades the poly-ubiquitin chain conjugated substrate proteins [12,14].

As the predominant protein degradation pathway in plants, the components of the UPS have been extensively characterized. The *Arabidopsis* genome encodes 2 E1 genes, 37 E2 genes, and approximately 1500 E3 genes [15–18]. Notably, E3 ligases exhibit remarkable diversity, with numerous families and members identified in plants. The substantial expansion of E3 genes in *Arabidopsis* and other plant genomes in general correlates with their crucial function in determining substrate specificity within the UPS pathway [15]. Consequently, E3 ligases are recognized as ubiquitous regulators that play critical roles in plant development and response to environmental changes [14].

E3 ligases, representing the most diverse proteins within UPS, can be classified into four subfamilies based on their characteristic functional domains and catalytic mechanisms: homology to E6-AP C terminus (HECT), really interesting new gene (RING), RBR (RING-in-between-RING), and U-box type E3 ligases [12,14,19]. Cullin-RING ubiquitin ligases (CRLs), which represent a prominent class of E3 ligases in *Arabidopsis*, belong to a subfamily of RING finger E3 ligases [20]. CRLs are multi-subunit complexes consisting of three major elements: a cullin scaffold protein, a RING finger protein that recruits E2 enzyme, and a substrate-recognizing module [20,21]. CRLs are subdivided into four well-characterized subfamilies based on the cullin isoform and the substrate-recognizing module: Cullin 1 (CUL1)-based, also known as S phase kinase-associated protein 1–CUL1–F-box; CUL3-based with bric-a-brac–tramtrack–broad complex substrate specificity subunits; CUL4-based with DNA damage-binding domain-containing substrate specificity subunits; and anaphase-promoting complex (APC) with the cullin-like protein APC2 [12,14]. These E3s are named for the CUL protein present with the substrate-binding subunit in superscript.

### Degradation of photomorphogenesis-promoting regulators by negative E3 ligases

**Figure 1:**
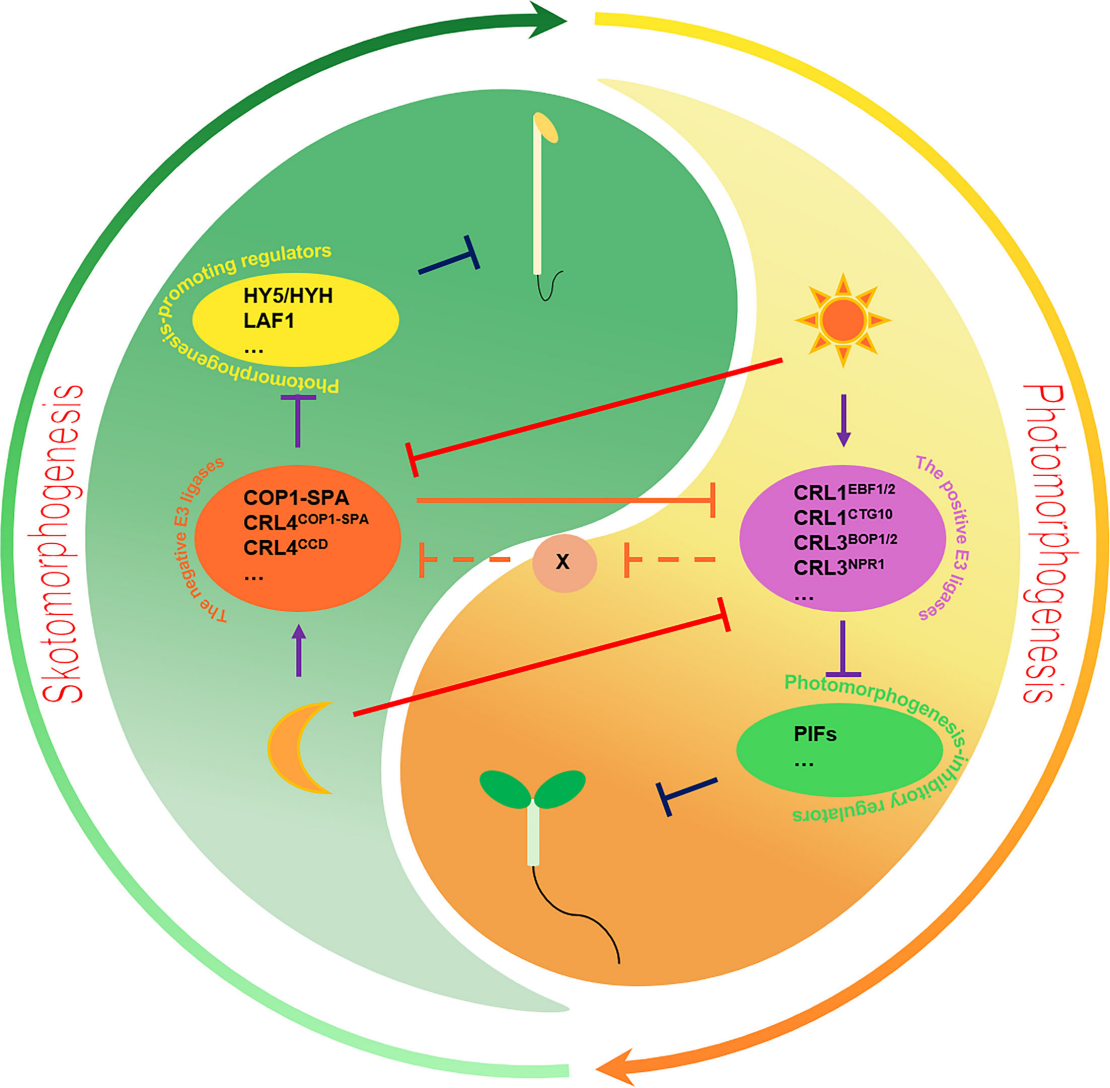
The ubiquitin–proteasome system in photomorphogenesis. In darkness, the negative E3 ligases, such as CRL4^COP1–SPA^, target photomorphogenesis-promoting regulators, particularly HY5, for ubiquitination and degradation, therefore repressing photomorphogenesis. Under light conditions, the positive E3 ligases like CRL1^EBF1/2^ promote the ubiquitination and degradation of photomorphogenesis-inhibitory regulators, including PIFs, ensuring appropriate seedling photomorphogenic development. Moreover, CRL4^COP1–SPA^ can target CRL1^EBF1/2^ for ubiquitination and degradation, thus affecting the stability of PIF3. Conversely, we put forward a hypothesis that an unidentified positive E3 ligase may positively regulate the stability of COP1 by modulating an intermediate E3 ligase X, which is capable of targeting COP1 for ubiquitination and degradation. In this model, positive E3 ligases are defined as E3 ligases that promote photomorphogenesis, as evidenced by the phenotypes of their mutants, whereas negative E3 ligases are those that repress photomorphogenesis. PIF, phytochrome-interacting factor.

The UPS serves as a crucial regulatory mechanism in photomorphogenesis, significantly influencing plant growth and development in response to the changing light conditions. CONSTITUTIVE PHOTOMORPHOGENIC1 (COP1), a RING type E3 ligase, was initially characterized as a key negative regulator of photomorphogenesis and subsequently as antagonistic to ELONGATED HYPOCOTYL5 (HY5), a core positive regulator of photomorphogenesis [1]. Under dark conditions, COP1 exhibits maximal activity, forming functional COP1–SUPPRESSOR OF PHYA-105 (SPA) E3 complexes through its interaction with SPA proteins [22]. Further, COP1–SPA complexes, serving as substrate receptors, can assemble into multimeric CRL4^COP1–SPA^ E3 ligase complexes [23,24]. These CRL4^COP1–SPA^ E3 ligase complexes target photomorphogenesis-promoting regulators, such as transcriptional factors (e.g. HY5, HY5 HOMOLOGUE (HYH), and LONG AFTER FAR-RED LIGHT 1 (LAF1)) and photoreceptors (e.g. phyA, phyB, cryptochrome 1 (CRY1), and CRY2), for ubiquitination and degradation through the UPS, thereby repressing photomorphogenesis in the dark (Figure 1) [1].

Another central negative complex in photomorphogenesis is the CUL4-based E3 ligase CRL4^CDD^, comprising three main components: COP10, damaged DNA binding protein 1, and de-etiolated 1 [25,26]. *COP10* encodes a small ubiquitin E2 variant protein, and both COP10 itself and the CRL4^CDD^ complex are known to enhance the activity of multiple E2 enzymes [25]. In darkness, the CRL4^CDD^ complex acting together with COP1 facilitates the ubiquitination and degradation of photomorphogenesis-promoting regulators, as well as LONG HYPOCOTYL IN FAR-RED 1 (HFR1), ensuring proper repression of photomorphogenic development (Figure 1) [25–27].

Unlike CRL4^COP1–SPA^ and CRL4^CDD^ E3 ligase complexes, the CUL3-based E3 ligases CRL3^LRBs^ and CRL3^NPH3^ function as negative regulators under high-intensity light conditions. Under strong red light, recruitment of CRL3^LRBs^ E3 ligases to phyB induces the polyubiquitination and degradation of phyB [28,29]. Similarly, CRL3^LRBs^ also mediates the polyubiquitination and degradation of CRY1 and CRY2 under high-intensity blue light [30,31]. Moreover, another CUL3-based E3 ligase, CRL3^NPH3^, targets phototropin1 for polyubiquitination and subsequent 26S proteasome-mediated degradation under high-intensity blue light [32]. The strong light-dependent ubiquitination and degradation of photoreceptors by CRL3^LRBs^ and CRL3^NPH3^ E3 ligases is thought to be beneficial for preventing over-stimulation and maintaining light-signaling homeostasis [9].

### Degradation of photomorphogenesis-inhibitory regulators by positive E3 ligases

Upon light exposure, light-activated photoreceptors inhibit the activity of COP1 through two distinct mechanisms: either by promoting the dissociation of COP1–SPA complexes or by facilitating COP1 migration to the cytosol [33–40]. This light-induced inactivation of COP1 leads to the stabilization and accumulation of photomorphogenesis-promoting regulators, particularly HY5, thereby initiating seedling photomorphogenic development. Meanwhile, a deubiquitinating enzyme Ub-SPECIFIC PROTEASE 14 deubiquitinates HY5 and protects it from degradation by 26S proteasomes under light [41]. Then, HY5, a bZIP-type transcription factor, directly binds to the promoters of light-responsive genes and promotes photomorphogenic development.

Concomitant with HY5 accumulation under light conditions, the degradation of phytochrome-interacting factor (PIF) proteins is initiated, thereby facilitating the onset of photomorphogenesis [42]. PIF proteins, belonging to the bHLH transcription factor family, serve as key repressors of photomorphogenesis. PIF1, PIF3, PIF4, PIF5, PIF7, and PIF8 positively regulate the hypocotyl elongation of *Arabidopsis* seedlings to varying degrees under red light, and strikingly, the higher-order *pifQ* (*pif1*/*2*/*3*/*4*) mutant displays constitutive photomorphogenesis in darkness, mimicking the phenotype of light-grown wild-type seedlings [42–44].

In *Arabidopsis*, multiple E3 ubiquitin ligases, including three characterized CRL types, CRL1, CRL3, and CRL4, have been identified to be involved in the light-induced ubiquitination and degradation of PIF proteins [20]. The CRL1^CTG10^ E3 ligase, formed by the F-box protein COLD TEMPERATURE-GERMINATING (CTG)-10 in complex with CUL1, negatively regulates PIF1 stability under light conditions (Figure 1 and Table 1) [45]. Two distinct E3 ligases, CRL1^EBF1/2^ and CRL3^LRBs^, target PIF3 for light-induced ubiquitination and degradation in different manners [28,46]. CRL1^EBF1/2^ functions as a negative E3 ligase and down-regulates the stability of PIF3 in response to different light intensities, thus promoting photomorphogenic development (Figure 1 and Table 1) [46]. The degradation of phyB-PIF3 by CRL3^LRBs^ E3 ligase under strong red light serves as a mechanism to attenuate light signaling and maintain light-signaling homeostasis (Table 1) [28]. Different E3 ligases are involved in the ubiquitination of PIF4 under various light conditions. CRL3^BOP1/2^ E3 ligase mediates the polyubiquitination and degradation of PIF4 under red light, while CRL3^NPR1^ directs PIF4 polyubiquitination and degradation in response to blue light (Figure 1 and Table 1) [47,48]. Additionally, CRL4^COP1–SPA^ can promote the ubiquitination and degradation of PIF1, PIF5, and PIF8 in the light to optimize photomorphogenic development in *Arabidopsis*. CRL4^COP1–SPA^ ubiquitinates PIF1 specifically during the dark to light transition, promotes the ubiquitination and degradation of PIF5 in response to red light, and preferentially targets PIF8 for ubiquitination and degradation in the dark (Table 1) [44,49,50].

**Table 1:** List of the E3 ligases that target PIFs for degradation

E3 ligase	Target protein	Light condition	Effect	Citation
CRL1^CTG10^	PIF1	Light	Germination	[45]
CRL1^EBF1/2^	PIF3	Different light intensities	Promoting photomorphogenesis	[46]
CRL3^LRBs^	PIF3	Strong red light	Attenuating light responses	[28]
CRL3^BOP1/2^	PIF4	Red light	Promoting photomorphogenesis	[47]
CRL3^NPR1^	PIF4	Blue light	Promoting photomorphogenesis	[48]
CRL4^COP1–SPA^	PIF1	Dark to light transition	Optimizing photomorphogenesis	[49]
CRL4^COP1–SPA^	PIF5	Red light	Optimizing photomorphogenesis	[50]
CRL4^COP1–SPA^	PIF8	Dark	Optimizing photomorphogenesis	[44]

### Interplay between negative and positive E3 ligases during photomorphogenesis

As the cycle of day and night is inevitable, plants are compelled to adapt to the constantly changing light conditions and adjust their developmental programs accordingly. The negative and positive E3 ubiquitin ligases modulate the stability of photomorphogenesis-promoting or inhibitory regulators, respectively, thereby ensuring proper skotomorphogenic growth in the dark and photomorphogenic growth in the light. Therefore, the interplay between the positive and negative E3 ligases is central to light signaling and plant development.

Light exposure triggers CRL1^EBF1/2^-mediated ubiquitination and degradation of PIF3 to promote photomorphogenic development (Table 1) [46]. However, in the dark, COP1 promotes the stability of PIF3 by targeting CRL1^EBF1/2^ for ubiquitination and degradation, thus repressing photomorphogenesis (Figure 1) [26,46,51]. This regulatory mechanism prompts an interesting question: could positive E3 ligases also modulate the stability of negative E3 ligases? Such a regulatory interaction would potentially introduce an additional layer of complexity to this regulatory network. Forward genetic screens for *cop1–6* suppressors have led to the identification of a RING-finger protein, COP1 SUPPRESSOR1 (CSU1), which functions as an E3 ligase for COP1 [52]. CSU1 targets COP1 for ubiquitination and degradation in dark-grown seedlings, thus maintaining COP1 homeostasis in darkness [52]. Intriguingly, *csu1* mutants exhibit shorter hypocotyls compared with wild type under light conditions [52]. This observation suggests that CSU1 acts as a negative regulator of photomorphogenesis in the light, implying that CSU1 may target other yet-to-be-identified factors to repress photomorphogenesis. Notably, the CSU1–COP1 module does not provide an answer to the aforementioned question. Thus, we propose a hypothesis: an as-yet-unidentified positive E3 ligase could positively regulate the stability of COP1 by modulating an intermediate E3 ligase X, which is capable of targeting COP1 for ubiquitination and degradation (Figure 1). It is hypothesized that CSU1 may be the potential E3 ligase X, given that it is currently the only identified E3 ligase known to target COP1 [52]. Thus, technologies such as IP–MS can be adopted to identify the as-yet-unidentified positive E3 ligase, which can be immunoprecipitated by both COP1 and CSU1. Nevertheless, this hypothesis awaits verification in future research endeavors.

Under UV-B light, the UV-B–inducible protein RUP1/RUP2 associates with the CUL4 scaffold to form E3 ligase CRL4^RUP1/2^, thus repressing photomorphogenesis by mediating the ubiquitination and degradation of HY5. Conversely, CRL4^COP1–SPA^ E3 ligase serves as a positive regulator under UV-B light and targets RUP1/RUP2 for ubiquitination and degradation under UV-B light, leading to the stabilization of HY5 [53]. Therefore, these two E3 ligases, CRL4^COP1–SPA^ and CRL4^RUP1/2^, act antagonistically in regulating the stability of HY5, thus ensuring the photomorphogenic development in response to UV-B irradiation.

## Discussion

### Photomorphogenesis-skotomorphogenesis

In the light signaling machinery of plants, photoreceptors act as the master control switches, perceiving light and determining whether photomorphogenesis is turned ‘on’ or ‘off.’ These photoreceptors, including phytochromes and cryptochromes, initiate downstream signaling cascades to regulate plant growth and development in response to light. Meanwhile, the UPS functions as a transverter, dynamically controlling the transition between skotomorphogenesis and photomorphogenesis by regulating the stability and activity of key signaling components. In darkness, photomorphogenesis is turned ‘off.’ In this case, the negative E3 ligases (e.g. CRL4^COP1–SPA^) target photomorphogenesis-promoting regulators (e.g. HY5) for ubiquitination and degradation, therefore repressing photomorphogenesis. Upon light perception, the light-activated photoreceptors inactivate the negative E3 ligases and thus stabilize the positive E3 ligases (e.g. CRL1^EBF1/2^) to promote the ubiquitination and degradation of photomorphogenesis-inhibitory regulators (e.g. PIFs), ensuring proper seedling photomorphogenic development [1,26].

The stability of PIFs, which are identified as the key negative regulators of photomorphogenesis, is also under the control of COP1. COP1 increases the stability of PIF1, PIF3, PIF4, and PIF5 in the dark, while light-induced phosphorylation of PIFs leads to subsequent ubiquitination and degradation of them [20,50,54,55]. It is well established that COP1 enhances the stability of PIF3 by targeting its E3 ligase EBF1/2 for ubiquitination and degradation [46]. However, the mechanisms by which COP1 stabilizes PIF1/4/5 remain unclear and require further investigation. It is speculated that COP1 may target other yet-to-be-identified E3 ligases for PIFs to stabilize PIFs. It is speculated that IP–MS technology can be employed to identify the potential E3 ligases that are capable of being immunoprecipitated by both COP1 and PIFs.

### COP/DET/FUS in light signaling

In *Arabidopsis*, a set of proteins collectively known as COP/DET/FUS have been shown to function as central regulators of photomorphogenic development, as their loss-of-function mutants initially identified in genetic screens display constitutively photomorphogenic phenotypes in darkness [26,56]. These proteins form three distinct protein complexes: CRL4^COP1–SPA^, CRL4^CCD^, and the COP9 signalosome (CSN). CRL4^COP1–SPA^ and CRL4^CCD^ function as E3 ligases and target photomorphogenesis-promoting regulators for ubiquitination and degradation, thus repressing photomorphogenesis. The CSN complex, serving as a cullin deneddylase, regulates the activity and assembly of CRLs [57]. To achieve a comprehensive understanding of how these three types of complexes act together at both biochemical and functional levels will present a significant challenge in the coming years.

### COP1 functions as a sole E3 protein or in the form of COP1–SPA and CRL4^COP1–SPA^ complexes?

COP1 is a RING-type E3 ligase and has been experimentally verified to ubiquitinate target proteins *in vitro* as the sole E3 protein [58]. Additionally, COP1 also performs its function in the form of COP1–SPA and CRL4^COP1–SPA^ complexes [56,58]. Hence, an open question remains: what factors determine when COP1/SPA acts alone or acts within CRL4^COP1–SPA^ complexes? It was reported that COP1 is also involved in light-induced degradation of PIF1, PIF3, and PIF8, indicating that COP1 has functional roles in the light as well [44,49,50]. It was hypothesized that COP1 may function in the light, either as COP1–SPA complex or CRL4^COP1–SPA^ supercomplexes. Deciphering the mechanisms by which COP1–SPA complex or CRL4^COP1–SPA^ supercomplexes recognize their respective substrates is another challenge.

### Non-proteolytic role of COP1

Interestingly, a recent study has revealed that COP1 can regulate photomorphogenesis through non-proteolytic ubiquitination, a previously unknown activity. COP1 directly interacted with GRETCHEN HAGEN 3.5 (GH3.5), a synthetase that conjugates amino acids to indole-3-acetic acid. This interaction promotes K63-linked polyubiquitination of GH3.5, leading to its inactivation and subsequently promoting hypocotyl elongation in the dark [59]. This finding raises new questions regarding how COP1, in COP1–SPA form or CRL4^COP1–SPA^ super complexes, recognizes specific substrates and executes distinct ubiquitination modifications.

Taken together, the UPS acts downstream of the complex and fluctuating light conditions, enabling plants to adjust their proteomes to the surrounding light environment for growth and survival. The number of genes encoding UPS components is substantial, and there is still much to be done to fully elucidate their functional roles. Further identification of distinct UPS components, such as E3 ligases and substrates of E3, that are involved in light signaling pathway will help to elucidate the molecular regulatory mechanisms that underpin plant responses to the fluctuating light conditions at the protein level. It can provide valuable strategies to fine-tune crop responses to light conditions through manipulating specific UPS components. For example, the *Gmcop1b* mutant generated by the CRISPR/Cas9 technology exhibits reduced shade response induced by low blue light and achieves a higher yield [60].

**Summary**

- In the light signaling machinery, photoreceptors act as the master control switches, perceiving light and determining whether photomorphogenesis is turned ‘on’ or ‘off.’ The UPS functions as a transverter, dynamically controlling the transition between skotomorphogenesis and photomorphogenesis.
- In darkness, the negative E3 ligases target photomorphogenesis-promoting regulators for ubiquitination and degradation, therefore repressing photomorphogenesis.
- Under light conditions, the positive E3 ligases promote the ubiquitination and degradation of photomorphogenesis-inhibitory regulators, ensuring proper seedling photomorphogenic development.
- The interplay between the positive and negative E3 ligases is central to light signaling and seedling photomorphogenic development.

## Open Access

This article has been published open access under our Subscribe to Open programme, made possible through the support of our subscribing institutions, learn more here: https://portlandpress.com/pages/open_access_options_and_prices#conditional

## Abbreviations

APC: anaphase-promoting complex
COP1: CONSTITUTIVE PHOTOMORPHOGENIC1
CRLs: Cullin-RING ubiquitin ligases
CSN: COP9 signalosome
CTG: COLD TEMPERATURE-GERMINATING
GH3.5: GRETCHEN HAGEN 3.5
HY5: ELONGATED HYPOCOTYL5
PIF: phytochrome-interacting factor
PTMs: post-translational modifications
RING: really interesting new gene
SPA: SUPPRESSOR OF PHYA-105
UPS: ubiquitin–proteasome system
